# Physician’s conceptions of the decision-making process when managing febrile infants ≤ 60 days old: a phenomenographic qualitative study

**DOI:** 10.1186/s12887-024-04548-x

**Published:** 2024-01-26

**Authors:** Ioannis Orfanos, Rose-Marie Lindkvist, Erik G. A. Eklund, Kristina Elfving, Tobias Alfvén, Tom J. de Koning, Charlotte Castor

**Affiliations:** 1 Department of Clinical Sciences, Lund University, Lund, Sweden; 2 Department of Pediatrics, Skåne University Hospital, Lund University, Akutgatan 4, 221 85 Lund, Sweden; 3 Department of Pediatrics, Sahlgrenska Academy, University of Gothenburg, Gothenburg, Sweden; 4 School of Public Health and Community Medicine, Sahlgrenska Academy, University of Gothenburg, Gothenburg, Sweden; 5 Department of Global Public Health, Karolinska Institute, Stockholm, Sweden; 6 Sachs’ Children and Youth Hospital, Stockholm, Sweden; 7 Karolinska Insitutet, Stockholm, Sweden; 8 Department of Health Sciences, Lund University, Lund, Sweden

**Keywords:** Febrile infants, Guidelines, Adherence, Lumbar puncture

## Abstract

**Background:**

The management of febrile infants aged ≤ 60 days and adherence to guidelines vary greatly. Our objective was to describe the process of decision-making when managing febrile infants aged ≤ 60 days and to describe the factors that influenced this decision.

**Methods:**

We conducted 6 focus group discussions with 19 clinically active physicians in the pediatric emergency departments of 2 university hospitals in Skåne region, Sweden. We followed an inductive qualitative design, using a phenomenological approach. A second-order perspective was used, focusing on how physicians perceived the phenomenon (managing fever in infants) rather than the phenomenon itself. The transcribed interviews were analyzed using a 7-step approach.

**Results:**

Performing a lumbar puncture (LP) was conceived as a complex, emotionally and mentally laden procedure and dominated the group discussions. Three central categories emerged as factors that influenced the decision-making process on whether to perform an LP: 1) a possible focus of infection that could explain the origin of the fever, 2) questioning whether the temperature at home reported by the parents was a fever, especially if it was ≤ 38.2°C, and 3) the infant’s general condition and questioning the need for LP in case of well-appearing infants. Around these 3 central categories evolved 6 secondary categories that influenced the decision-making process of whether to perform an LP or not: 1) the physicians’ desire to be able to trust their judgement, 2) fearing the risk of failure, 3) avoiding burdensome work, 4) taking others into account, 5) balancing guidelines and resources, and 6) seeing a need to practice and learn to perform LP.

**Conclusions:**

The difficulty and emotional load of performing an LP were important factors that influenced the decision-making process regarding whether to perform an LP. Physicians highlighted the importance of being able to rely on their clinical judgment and make independent decisions. Guidelines may consider allowing a degree of flexibility and independent thinking to take into account patients’ characteristics and needs.

## Introduction

The management of young febrile infants aged ≤ 60 days has been the subject of extensive research in recent decades, and several guidelines have been developed to aid clinical investigation and treatment of these infants [1–5]. Management guidelines have been shown to improve the care of febrile infants, enhance patient safety, optimize resource utilization, and decrease variability in treatment and cost [6–10]. However, guidelines are often not applied in daily clinical practice and often takes time to be endorsed [11, 12]. Barriers to the implementation and use of guidelines have been investigated, and several possible etiologic factors have been identified. These factors can be divided into 3 major categories [13, 14]. The first category is related to physicians’ knowledge (e.g., awareness, familiarity) and attitudes (e.g., learning culture, agreement, motivation, skills). The second is related to guidelines (e.g., evidence, complexity, layout, applicability, and accessibility). Finally, the third category is related to external factors (e.g., workload, time restriction, lack of resources) [13, 14].

Despite the potential benefits of guidelines for febrile infants, numerous studies have shown great variation in the management of febrile infants and poor adherence to such guidelines [15–19]. Quantitative studies on management and prevalence showed that well-appearing febrile infants, infants without fever during the examination, or febrile infants seen in private pediatric offices underwent fewer investigations and therapeutic interventions than recommended [16, 20–22]. Hence, researchers hypothesized that the general condition of the infant, the absence of fever during the physical examination, and the physicians’ experience might influence the decision not to follow guidelines [16, 17]. Aronson et al., in a study with semi-structured interviews, aimed to learn the factors that influenced physicians’ decision to perform a lumbar puncture (LP). The factors that mostly emerged were physicians’ clinical experience, knowledge of newer recommendations and their supporting evidence, and personal values such as risk aversion or inclination for shared decision-making with the parents [23].

However, there is limited knowledge regarding the decision-making process itself. Also, it is needed a broader understanding of physicians’ perspectives and how they decide to follow guidelines when managing febrile infants. Investigating clinicians’ perspectives on decisions about the management of febrile infants might help to understand the contributing factors and potential barriers to using guidelines. This understanding may subsequently aid in guideline improvement and implementation. In particular, the management of febrile infants aged ≤ 60 days is a topic of ongoing debate, with various recommendations and reported variations.

Our objectives were:1) to describe the process of decision-making when managing febrile infants aged ≤ 60 days and 2) to describe the factors that influenced this decision.

## Methods

### Setting

The study was conducted at Skåne University Hospital, based in Sweden’s southernmost region, which includes 2 pediatric hospitals. These hospitals are located in 2 neighboring cities and have separate pediatric emergency departments (PEDs), with approximately 35 000 annual visits. The total catchment area is approximately 700 000 inhabitants.

These PEDs are staffed by pediatric residents and residents of general medicine, infectious diseases, and emergency medicine for 4–12 weeks of pediatric rotation. During office hours.

PEDs are supervised by pediatric specialists who belong to the Department of Pediatric Infectious Diseases and Emergency Pediatrics. Outside office hours the PEDs are supervised by on-call pediatric consultants accessible by telephone and are ready to attend physically. The on-call consultants practice in different pediatric subspecialties (i.e., endocrinology, gastroenterology, neurology, nephrology, and infectious diseases). Every year, around 500 infants ≤ 60 days of age with fever as the chief complaint present to these 2 PEDs, of which approximately 20% are ≤ 21 days of age. A local guideline for the management of febrile infants ≤ 60 days with fever without a source (FWS) was introduced in 2018, based on the “step by step” approach [3]. Among the recommendations are LP, urine and blood cultures, and admission with parenteral antibiotics for all ill-appearing infants and infants ≤ 21 days. For the well-appearing infants 22–60 days, it recommends blood tests (procalcitonin, C-reactive protein), urine dipstick, and specific actions according to the results.

### Study design

In this study, we followed an inductive qualitative design. We used a phenomenographic approach according to Sjöström and Dahlgren [24] to gain insight into physicians’ decision-making processes, including perceived barriers, facilitators, and motivators when managing febrile infants aged ≤ 60 days. Phenomenography takes a second order perspective by focusing on how the phenomenon (managing fever in infants) was perceived by physicians, rather than the phenomenon itself [24]. The need for an ethics approval of this study was deemed unnecessary according to Swedish legislation [25].

### Participants

All pediatric residents and specialists clinically active at the PED were invited to enroll during staff meetings and through follow-up emails. A letter describing the scope and aims of the study was sent to all. For data collection, we chose focus groups with physicians working in the same role at the PED to create an opportunity for better interaction between interviewees and to ensure better homogeneity [26–28]. Thus, the participants were divided into focus groups according to function (on-duty physically present at PED or on-call). The doctors on duty were either pediatric resident doctors or pediatricians who had recently completed their pediatric training. The on-call doctors were senior pediatric consultants. Verbal informed consent was obtained from all the participating physicians. The initial design was for groups of 4 to 6 participants. Few physicians did not show up or cancelled with a very short notice. Nineteen physicians participated in this study divided in 6 focus groups of 2 to 5 participants. The physicians varied in age, years of experience working with children, and number of febrile infants ≤ 21 days managed per month (Table 1).

**Table 1:** Composition of the focus groups and characteristics of the participants

	On-duty	On-call
Participants	11	8
Group 1	3	2
Group 2	4	2
Group 3	4	4
Female	7	5
Age (years)		
21–30	3	–
31–40	8	–
41–50	–	4
> 50	–	4
Years of working with children		
0–3	2	–
4–5	5	–
6–9	4	–
> 10	–	8
Febrile infants ≤ 21 days managed per month		
0–2	7	2
3–5	1	3
> 5	3	3

### Data collection

Focus group discussions were led by one co-author with support from another co-author with clinical experience in working as a pediatric nurse. Both have experience in conducting qualitative research and none was involved in the care of infants. Oral consent was obtained before the focus group discussions, and the participating physicians filled out a questionnaire containing information regarding their demographics and professional experience. The discussions were guided by a short topic list (Table 2), with follow-up questions based on responses for further exploration to yield the best possible information. The topics for the interviews were decided based on the main recommendations of the local guideline, on commonly voiced objections by physicians during the last years, on findings from quantitative studies on management variation in febrile infants aged ≤ 60 days, and questions used in similar previous studies. The focus group discussions were performed in April and May 2022 at local hospital facilities. They were audio recorded without mentioning the name or role of any of the participants, and were transcribed verbatim by a professional transcription service. The data transcription company follows strict General Data Protection Rules (GDPR) procedures.

**Table 2:** Topics and questions for focus group interviews

Topic	Example questions
Overall process of decision-making	What process of decision-making do you follow when managing fever in an infant (< 21 days)?
External factors affecting decision-making	What factors affect your decisions and how? (The general condition of the infant? Input from colleagues? Wishes and needs from parents?)
Decisions on diagnostics and treatment	What factors are crucial in decisions on diagnostic procedures (such as lumbar puncture, blood, and urine culture), treatment (such as antibiotics and hospitalization), and how?

### Analysis

The audio-recorded and transcribed interviews were analyzed using a stepwise phenomenographic approach, where physicians’ different perceptions of the phenomenon were identified and grouped into categories and conceptions. We followed a seven-step approach as described by Sjöström and Dahlgren (2002) [24]. In Step 1 we read the transcripts to get familiarized with the data. In Step 2, we identified the significant elements related to the aim. In Step 3, we condensed and identified the central parts. In Step 4, we performed a preliminary grouping of the central parts. In Step 5 we compared and revised the preliminary groups. In Step 6, we named the categories according to their essence. Finally, in step 7, we performed a contrastive comparison of categories and identified the uniqueness and resemblance between the categories. The second part of the analysis was to orient the categories toward intrinsic and extrinsic motivators known to influence the decision making -process [29]. Two of the coauthors conducted the primary analysis. Subsequently, the analysis was reviewed, revised, and confirmed in collaboration with another co-author. Finally, the results were discussed with all coauthors until a joint agreement and consensus was reached. Representative quotes were used to support and exemplify categories and are presented in Tables 3 and 4. The data were managed using NVivo software [QSR International Pty Ltd. (2018) NVivo (version 12]).

**Table 3:** Central categories which influence physicians’ decision-making process on whether to perform a lumbar puncture or not in febrile infants ≤ 60 day and relevant quotes

**Actively looking for a focus of infection**	
(on-call physician, focus group 5)	“If you find an obvious explanation; the infant has a cold, mom has a cold, someone else close by obviously has a cold. Then you may put yourself at ease if the child is well-appearing.”
**Questioning the fever**	
(on-duty physician, focus group 6)	“If you at some point are in a situation where the baby had fever at home, presents with no fever and [you think]: “What was that? Was it a thermometer that did not work? Was the baby warm because of how it was dressed?” You want to find an explanation as to why it might have been different at home and a normal temperature here.”
**Considering the general condition**	
(on-duty physician, focus group 3)	“I perceived that child as extremely healthy and content, and without any signs of meningitis. At that point, it would be a pity to start up the whole process (LP)”

**Table 4:** Intrinsic and extrinsic factors and motivators that influence physicians’ decision-making process on whether to perform a lumbar puncture and relevant quotes

Being driven by intrinsic motivators		Being driven by extrinsic motivators	
**Trusting own judgment**		**Taking others into account**	
a. On-call physician (focus group 1) b. On-call physician (focus group 4) c. On-duty physician (focus group 6) d. On-duty physician (focus group 5)	“A lot is positive with the guideline, but it is not possible to have a guideline which covers all scenarios” “You get very focused on the guideline instead of trying to get the feeling of the patient, like “How sick is this child?” And I think that is a bit dangerous.” “I feel safe in my medical judgement. I do not have too much anxiety about this baby because I have built up references. I can see that this, okey, this is a sick baby. Then I pull out the LP-tray.” “We had few babies which were considered well-appearing and were admitted for observation, and they had meningitis”	g. On-duty physician (focus group 2) h. On-duty physician (focus group 6) i. On-duty physician (focus group 3)	“You know, I don’t want to do anything unnecessary to the baby (…) And I do not want to worry parents who do not need to get worried.” “There are nurses with 25 years of experience, and you arrive as new and you do not have the clinical gaze. Then you may have a dialogue. You know, let’s do this. I always tend to ask ‘Well, what do you think?’” “And then you present it like it was not a sick baby and then they might say “Well, then you do not need to perform LP.”
**Fearing risk or failure**		**Seeing a need to practice and learn to perform LP**	
e. On-call physician (focus group 5)	“I guess, it’s better to perform LP on ten, than to miss one meningitis.”	j. On-call physician (focus group 5)	“It is better to perform LP, to just do it, and then the physician on duty will also learn and connect the dots: “Okey, this baby looks like this, nothing was growing. Then I had this baby where it still turned into meningitis.” You see, it is their way of building experience.”
**Avoiding burdensome work**		**Balancing guidelines and resources**	
f. On-call physician (focus group 5)	“To follow the protocol, creates more resistance if it concerns burdensome things which we physicians must do.”	k. On-duty physician (focus group 6)	“Even if you yourself don’t have much against doing it, it sort of becomes. It’s a big thing. There is a lot of staff involved, it takes time, it takes space.”

## Results

Physicians described the management of febrile infants as a process of considering several different parameters. Most of the process, particularly when to order urine or blood test, was described as relatively straightforward. However, the decision on whether to perform an LP was conceived as complex and dominated the discussion in all groups. The decision-making process on whether to perform an LP was often described as starting and subsequently evolving around three central categories. The first was actively looking for a focus of infection that could explain the origin of the fever. Signs or symptoms of upper respiratory tract infection were sought in the history or the physical examination. Also, information such as an ongoing viral infection in the family could be considered sufficient as a likely cause of the fever (Table 3).

The second central category was questioning the presence of the fever in the first place. Measurement of temperature at home which was not higher than 38.2 °C was at times not regarded as a fever, especially in cases where the infant was afebrile in the emergency department (Table 3). The third central category was considering the infant’s general condition. The physicians stated that they estimated the likelihood of meningitis to be very low and reasoned that LP was not motivated in cases of well-appearing infants (Table 3).

**Fig. 1:**
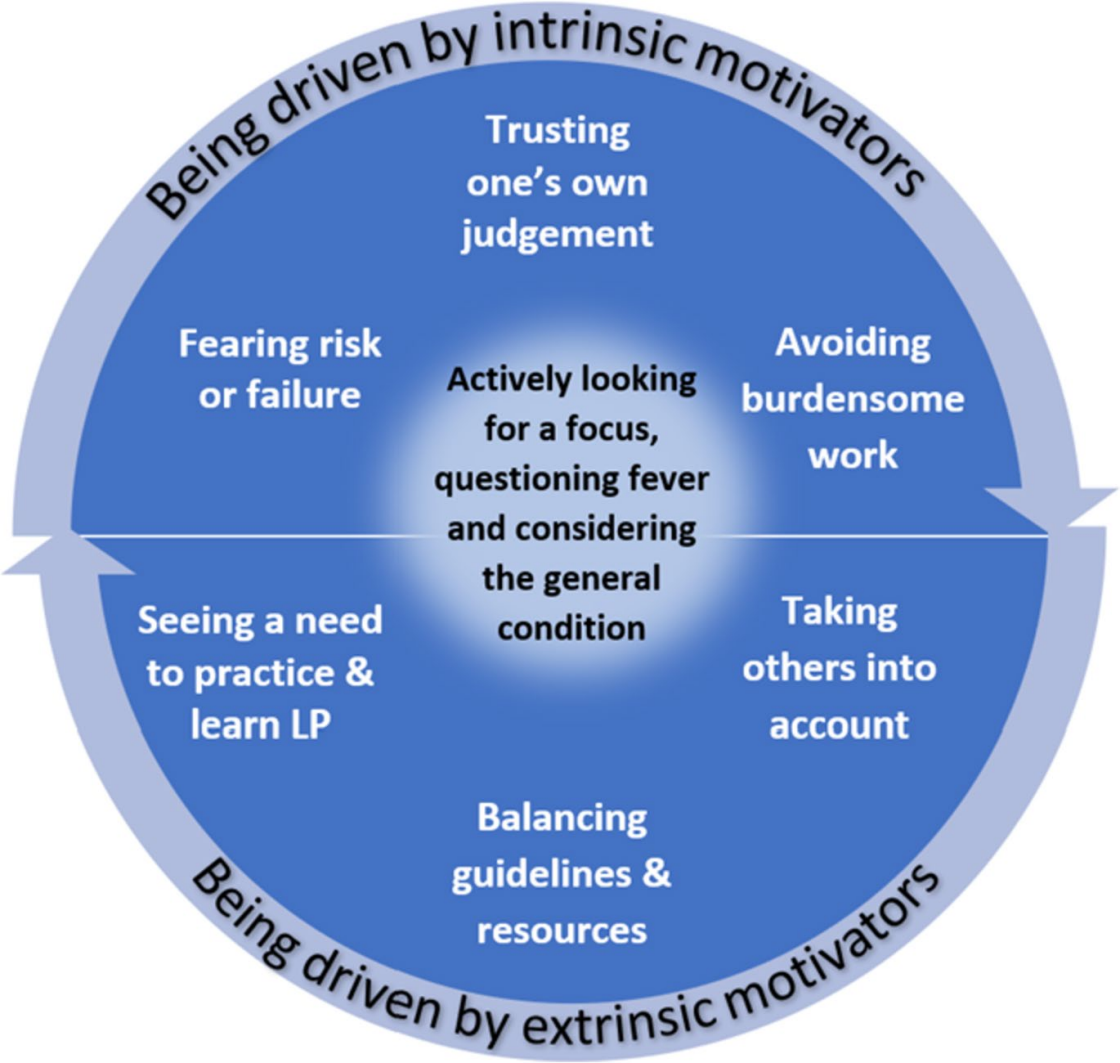
Central and secondary categories that influence the decision-making process on whether to perform an LP

Around these 3 central categories evolved 6 secondary categories, which influenced the decision-making process on whether to perform an LP (Fig. 1). We subsequently attributed these 6 secondary categories to 2 different motivators: “Being driven by intrinsic motivators” and “Being driven by extrinsic motivators”, which could give further insight into the physicians’ decision-making process and justification on whether to perform an LP. The circular shape and arrows in Fig. 1 illustrate how intrinsic and extrinsic motivators influence each other and are not isolated factors in the decision-making process.

### Trusting one’s own judgement

Physicians emphasized the need and sense of obligation to maintain professional responsibility in terms of not allowing guidelines to limit their ability to think and make well-informed decisions. Learning to trust their clinical judgment was described as an important part of practicing medicine and mentoring young colleagues. It was considered a matter of principle for both on-duty and on-call physicians to make decisions and to be confident with the decision made. It was discussed that guidelines cannot cover all clinical scenarios, and they are rather aimed at guiding and supporting decisions taken by the physician in charge (Table 4a). It was also mentioned that on one hand guidelines provide valuable help to inexperienced physicians, but on the other hand physicians need to learn and develop a way of working to prevent pitfalls that might arise by blindly following guidelines. Hence, the feeling of skipping liability by following guidelines was perceived to come with a risk of becoming a non-thinker (Table 4b). Physicians reasoned on whether to perform an LP by putting weight on their own experience and clinical judgement and whether this had played out well in previous similar situations (Table 4c). However, some physicians expressed less confidence in their clinical judgement and reluctance to skip the LP, since they recalled cases when they thought that the infant was well-appearing and turned out to have meningitis (Table 4d).

### Fearing risk or failure

The decision-making process regarding LP was described as both associated with the fear of missing a meningitis case with a potentially lethal outcome and the fear of not successfully performing LP. Physicians recalled previous experiences of cases that went wrong, which created a strong sense of risk aversion and zero tolerance for missing a single meningitis case (Table 4e). Physicians also referred to the effect of personality characteristics, where an anxiety-driven individual was less prone to skipping an LP if recommended by the guideline. Furthermore, physicians expressed anxiety about failing with the procedure of the LP. This anxiety was amplified in cases where parents were reluctant to give consent, and the physicians had to put effort into convincing them of the importance of LP.

### Avoiding burdensome work

Some physicians described LP as a mentally and emotionally laden intervention for themselves, the nurses, and the parents. Additionally, LP was described as a burdensome procedure requiring time and personnel in an already overwhelmed and understaffed PED. All of the above were perceived as factors that could potentially increase the reluctance to perform an LP. In contrast, decisions on procedures performed by nurses, such as obtaining blood samples or inserting intravenous cannulas, were much easier to make (Table 4f).

### Taking others into account

The views and perspectives of the parents were described as factors that decreased the motivation to perform an LP, since it was considered quite stressful for the parents (Table 4g). In addition, the advice and opinions of experienced nurses were described as valuable support for decisions on LP (Table 4h). A nurse could be quite worried about a baby where the physician was not and vice versa. Input from nurses was perceived to weigh more in situations where the physician was already reluctant to perform LP. More specifically, physicians described being inclined to do less in cases in which a nurse would strongly question the need to perform an LP. However, physicians emphasized their responsibility and role as decision-makers. On-duty physicians described sometimes asking for advice from on-call physicians to get support for an already taken decision on LP to share responsibility for not following the guideline. This was conceived to at times involve presenting cases in a somewhat angled way, emphasizing the clinical aspects that supported the decision not to perform an LP (Table 4i).

### Seeing a need to practice and learn to perform LP

On-call physicians brought forward the potentially problematic aspect of younger colleagues rarely performing an LP, which has the risk of gradually decreasing competence. Physicians stated that it is important for healthcare staff to learn to perform an LP and to continue practicing it (Table 4j).

### Balancing guidelines and resources

Physicians described that guidelines do not consider the conditions and available resources at the emergency department and how challenging it can be to perform LP in situations of high workload (Table 4k). In such situations, own judgement and input from other staff members were perceived to steer how working time is used and prioritized.

## Discussion

We performed an inductive qualitative study to gain insight into physicians’ decision-making process when managing infants with fever without source and to understand the factors that influenced this decision.

Quite interestingly, the group discussions evolved almost solely around the decision on whether to perform an LP, and much less about other aspects of the guidelines, such as blood tests, antibiotic treatment, or hospitalization. This is not surprising since it has been reported that LP is an emotionally laden procedure for health personnel and parents, and is distressful and painful for infants [30–32]. In our study, LP was described as burdensome and technically difficult. Fear of failure was often mentioned, and previous studies have shown up to 35% rates of failure when performing LP [33, 34]. Physician-related barriers, such as lack of skills and self-efficacy, have been highlighted as important factors by other studies as well [14]. Furthermore, physicians stated that LP is time- and resource-demanding, since you cannot do the procedure just by yourself, which adds additional workload to the already understaffed and overwhelmed emergency department. Heavy workload, time restriction, and lack of resources are all known external factors that act as barriers to following guidelines [14, 32, 35]. In addition, physicians expressed concern that LP is particularly stressful for parents, who often do not consent to the procedure. This is a finding identified by other studies as well [23, 31]. Physician’s attitudes toward shared decision-making and consideration of parents’ preferences are also among previously reported physician-related facilitators and barriers that influence the decision to follow guidelines [14, 23, 32].

We identified that the decision-making process started most often by evaluating the general medical condition, considering whether the infant had a fever, and trying to identify a possible focus of infection. Participating physicians described that they are much less inclined to perform LP if an infant is well-appearing, if there are indications of an upper respiratory tract infection in the infant or in a family member, or if the infant had low-grade fever at home (< 38.2 °C) and is afebrile at the PED. This is in line with several studies on the management of febrile infants that have shown that well-appearing infants, infants afebrile at the PED, and infants with respiratory symptoms undergo fewer investigations and procedures [16, 20–22]. This observation led researchers to hypothesize that these clinical parameters might influence physicians’ decisions not to follow the recommended management guideline [16–18, 36]. Our study supports this hypothesis and shows that the general condition, presence of fever, and suspected focus of infection seem to play a central role in physicians’ decision-making process to adhere to certain steps of the guideline.

We found that around the 3 central categories 6 secondary factors evolved that influence the decision-making process on whether to perform an LP. One is the physician’s clinical experience. In our study, young and/or inexperienced physicians appeared to express worries on whether they can rely on their clinical judgment, and they described that they are more inclined to follow the recommendation to perform LP. On the other hand, senior physicians expressed more confidence in their decision not to perform LP. A study by Pantell et al. also showed that experienced pediatricians in private offices often did not follow the guidelines and Aronson et al. also reported similar findings [16, 32].

Interestingly, physicians’ experiences appeared to influence the decision-making process in different and sometimes contradictory ways. Senior physicians working in the infectious disease department described themselves as more risk-averse and more inclined to perform an LP because they had managed infants with meningitis and had seen the dire consequences of this disease. In contrast, senior physicians from other pediatric subspecialties described being less inclined to perform an LP. They explicitly reasoned that their way had worked well so far, since they had not been involved in the management of infants with meningitis.

Both on-duty and on-call physicians mentioned the ability for clinical reasoning and decision-making as another important factor influencing the decision-making process. A possible limitation of guidelines is that they discourage independent and critical thinking, which can consequently affect physicians’ clinical reasoning and diagnostic capability [37]. This may in turn jeopardize patient safety if the physicians fail to recognize patients’ individual needs and preferences [37–39]. In addition to patient harm, restriction of independent thinking and decision-making can be harmful to physicians as well. Depression and burnout have been increasing among physicians, while empathy has been declining [40]. Physicians, as human beings, are dynamic, thoughtful, emotional, and finite. Acknowledging this could prevent creating working environments that contribute to physicians’ increasing struggle [41]. It has been demonstrated that autonomy and a sense of competence are important facilitators and motivators for physicians’ personal development and well-being [42, 43].

Our study contributes new insight into why physicians choose not to follow guidelines for febrile infants and that the decision whether to perform LP or not has a central role in the decision-making process. We describe that the decision-making process follows a step-wise approach, starting by considering whether the infant has a fever or not, evaluating the clinical appearance, and searching for a focus of infection that could explain the fever. The information derived from these 3 central reasoning categories is often used to justify omitting LP, even if it is recommended by the guideline. Subsequently, the decision-making process regarding whether to perform an LP is influenced by 6 factors that weigh differently between on-duty and/or inexperienced versus on-call and/or experienced physicians. Among these factors, the difficulty and emotional load of LP, balancing workload, and desire for autonomy in clinical reasoning were perceived as central factors in the decision-making process. This new knowledge can act as a framework to target important elements for a more successful guideline implementation process. Training on performing LP, written instructions, and checklists could increase competence, shorten the duration of the procedure, and mitigate the fear of failure. Better parent information materials, such as pictographs, leaflets, and mobile applications, could decrease the mental and emotional load of LP and improve the shared decision-making process between doctors and parents [32, 44]. Additionally, guidelines should allow a degree of flexibility, independent thinking, and consideration of patients’ characteristics and needs.

## Limitations

Our study has several limitations. First, our initial plan was to construct groups of 4 to 8 participants. Due to work-related constraints, few groups had only 2 or 3 participants, which might have prohibited a freer expression of opinions. However, the themes were quite similar between the groups despite the number of participants. Second, the study was conducted in two university hospitals within one trust; thus, the perspectives of physicians might not be transferable and do not necessarily reflect the opinions of physicians in other non-academic hospital settings. Third, we chose an inductive qualitative design and encouraged the participants to express their thoughts about managing febrile infants; the interviewers avoided intervening and steering the discussions too much. Thus, the discussions evolved mostly around the LP and did not allow us to draw conclusions about the decision-making process regarding the other components of the guidelines. Finally, when analyzing, categorizing, and prioritizing perceptions, there is always the risk of misinterpretation and questioning “if it is the chicken or the egg that came first”. We interpreted and concluded that the decision-making process started with 3 major categories and subsequently evolved to 6 secondary categories. However, it cannot be excluded that one of the secondary factors, such as the mental and emotional load of the decision to perform an LP or the workload of the procedure, could be the decisive factors that triggered the decision-making process to try to find a focus of infection, question the presence of fever, or rely on a good general condition to exclude the possibility of meningitis. Studying such relationships requires more in-depth interviews as a follow-up to the present study.

## Conclusions

The decision on whether to perform LP played the most central role in the decision-making process when managing febrile infants. The general appearance, presence of fever, and possible focus of infection were the primary factors that influenced physicians to omit LP. The difficulty and emotional load of performing LP was an important barrier to following guidelines. Physicians highlighted the importance of being able to rely on their clinical judgment and make independent decisions. Guidelines may consider allowing a degree of flexibility and independent thinking to take into account patients’ characteristics and needs.

## Data Availability

Data are available from the corresponding author upon request.

## Abbreviations

LP: Lumbar puncture
PED: Pediatric Emergency Department
FWS: Fever without a source
